# Interpretive time-frequency analysis of genomic sequences

**DOI:** 10.1186/s12859-017-1524-0

**Published:** 2017-03-22

**Authors:** Hamed Hassani Saadi, Reza Sameni, Amin Zollanvari

**Affiliations:** 1 School of Electrical and Computer Engineering, Shiraz University, Shiraz, Iran; 2 Department of Electrical and Electronic Engineering, Nazarbayev University, Astana, Kazakhstan

**Keywords:** Genomic signal processing, Time-frequency analysis, Interpretive signal processing

## Abstract

**Background:**

Time-Frequency (TF) analysis has been extensively used for the analysis of non-stationary numeric signals in the past decade. At the same time, recent studies have statistically confirmed the non-stationarity of genomic non-numeric sequences and suggested the use of non-stationary analysis for these sequences. The conventional approach to analyze non-numeric genomic sequences using techniques specific to numerical data is to convert non-numerical data into numerical values in some way and then apply time or transform domain signal processing algorithms. Nevertheless, this approach raises questions regarding the relative magnitudes under numeric transforms, which can potentially lead to spurious patterns or misinterpretation of results.

**Results:**

In this paper, using the notion of interpretive signal processing (ISP) and by redefining correlation functions for non-numeric sequences, a general class of TF transforms are extended and applied to non-numerical genomic sequences. The technique has been successfully evaluated on synthetic and real DNA sequences.

**Conclusion:**

The proposed framework is fairly generic and is believed to be useful for extracting quantitative and visual information regarding local and global periodicity, symmetry, (non-) stationarity and spectral color of genomic sequences. The notion of interpretive time-frequency analysis introduced in this work can be considered as the first step towards the development of a rigorous mathematical construct for genomic signal processing.

## Background

The application of signal processing techniques in genomics has found a great deal of attention and applications in the past decade [1–4]. Nevertheless, an important class of analytical tools in signal processing that have not been yet fully formulated in genomics is the class of joint time-frequency (TF) distributions and transforms. These are powerful mathematical tools with various applications in signal processing [5, 6].

The major advantage of TF transforms and distributions over conventional Fourier analysis is to simultaneously retrieve the temporal (or spatial) and frequency domain structure of non-stationary data. In other words, while the temporal evolution of the frequency contents of a signal is lost in the conventional spectral estimation using the Fourier analysis, the TF gives a detailed view of such information for non-stationary signals. At the same time, several studies have statistically confirmed the non-stationarity of genomic sequences and suggested the use of non-stationary analysis for these sequences [7–9]. There could be many potential applications by applying the TF transforms to genomic sequences. Nevertheless, the first step in this line of work is to be able to apply these transforms to non-numerical genomic sequences.

The conventional approach to analyze genomic sequences using techniques specific to numerical data is to convert non-numerical genomic data into numerical values in some way and then apply time or transform domain signal processing algorithms to the resulting numeric series [10, 11]. Despite the promising results achieved by these methods, the procedure of converting genomic data into numerical data has been the bottleneck for these techniques—there is no concrete one-to-one map between non-numeric data and the numeric domain. Moreover, the process of converting non-numeric data to numeric values, can have misleading outcomes. For instance, the genomic alphabet (A, G, C and T) *is not an ordered set*. However, in mapping this alphabet to real values, the sequence is implicitly mapped to an ordered set, which raises expectations regarding their relative magnitudes under numeric transforms, resulting in misinterpretation of the processing results.

In [12], we introduced the notion of *interpretive signal processing* (ISP) as a novel approach for extending signal processing algorithms to non-numerical data. ISP can be seen as a subset of the general notion of *sequential pattern mining*, which has become a prominent topic in sequence data mining during recent years. In ISP, instead of coding non-numerical strings into numerical ones, the basic idea is to use the interpretations of conventional signal processing algorithms to reconstruct similar techniques that are directly applicable to non-numerical data. The notion of ISP is fairly general and may be used in various applications.

In this study, we employ ISP for applying a general class of TF transforms to analyze genomic sequences. As in real valued signals, the advantage of TF analysis over basic Fourier analysis is that it provides a means of analyzing both local and global patterns within a non-stationary sequence. Therefore, using TF transforms, local (yet significant) events which are commonly dominated by averaging in classical Fourier analysis can be identified within a sequence. As a proof-of-principle, we use the ISP-inspired TF analysis for detecting periodicities in coding regions and also detecting repetitive sub-sequences, also known as *tandem repeats*. The length and period of such sequences have important biological implications and several methods have been presented in the past for detecting these sub-sequences [11, 13, 14].

The rest of the paper is organized as follows. We first review the basics of a general class of time-frequency transforms. Then the general idea of ISP is illustrated with a simple example. The extension of time-frequency transforms for non-numeric genomic data is presented next, followed by some concluding remarks and future perspective of the work.

## Methods

### Joint time-frequency analysis

We first review some general concepts of bilinear TF analysis that are later extended to non-numerical data.

Correlation is a primary concept of great value in most signal processing algorithms. The *instantaneous cross-correlation* of the signals *x*(*t*) and *y*(*t*) is defined

1 $$ r_{xy}(t,\tau) = x\left(t-\frac{\tau}{2}\right)y^{*}\left(t+\frac{\tau}{2}\right)   $$

which for real-valued signals is a simple measure of similarity of *x*(*t*−*τ*/2) and *y*(*t*+*τ*/2). In fact, in (1), the multiplication operator is used to measure the similarity of its operands. We will later show how this product can be replaced by the values of the similarity matrix of the genomic “alphabets”.

By summing *r*_*xy*_(*t*,*τ*) (or integrating for continuous-time signals), the *cross-correlation function* is achieved.

2 $$ R_{xy}(\tau) = \sum_{t=-\infty}^{\infty}r_{xy}(t,\tau)   $$

This function is a measure of the *average similarity* of the two signals with *τ*-samples of time lag. Alternatively, by summing *r*_*xy*_(*t*,*τ*) over *τ*, a measure of *signal symmetry* is achieved.

3 $$ s_{xy}(t) = \sum_{\tau=-\infty}^{\infty}r_{xy}(t,\tau)   $$

Taking the Fourier transform of *R*_*xy*_(*τ*) with respect to *τ*, results in the *cross-spectrum*. 
4


where  represents the Fourier transform.

The *cross-Wigner-Ville* is the Fourier transforms of *r*_*xy*_(*t*,*τ*) with respect to *τ*. 
5


The Wigner-Ville (WV) transform can be interpreted as the time-variant extension of (4), which is specifically useful for the spectral study of non-stationary signals.

The last TF transform that we introduce is the *ambiguity function* (AF), defined as follows 
6


**Fig. 1:**
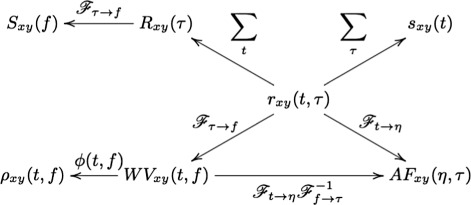
Summary of bilinear Time-Frequency transforms inter-relationship

The ambiguity function is basically a *time-frequency correlation* with a maximum at the origin [5]. It has been shown that the ambiguity function can be used to discriminate signals with different spectral color and temporal correlation [5]. The relationship between the instantaneous correlation and the other bilinear transforms is summarized in Fig. 1.

Due to the *bilinear form* of the WV transform (containing the product of *x*(·) and *y*(·)), undesired cross-terms appear in the time-frequency plane. The cross-terms can be attenuated by filtering the WV transform using a TF *kernel* *ϕ*(·,·), which results in the following general form

7 $$ \rho_{xy}(t,f) = {WV}_{xy}(t,f) ** \phi(t,f)   $$

where ∗∗ is the two-dimensional convolution operator. Equation (5) is the most general form of a general class of TF transforms (or TF *distributions*) known as the *Cohen class* [15]. The properties of these distributions are controlled by *ϕ*(*t,f*). According to Fig. 1, *ϕ*(*t,f*) may also be applied to the TF transform in the Ambiguity plane, where it takes a multiplicative form rather than two-dimensional convolution.

Equations (1)–(7) can be calculated for a single signal by setting *y*(*t*)=*x*(*t*), which gives the similarity of a signal with its time-lagged variants in the time and frequency domain.

**Fig. 2:**
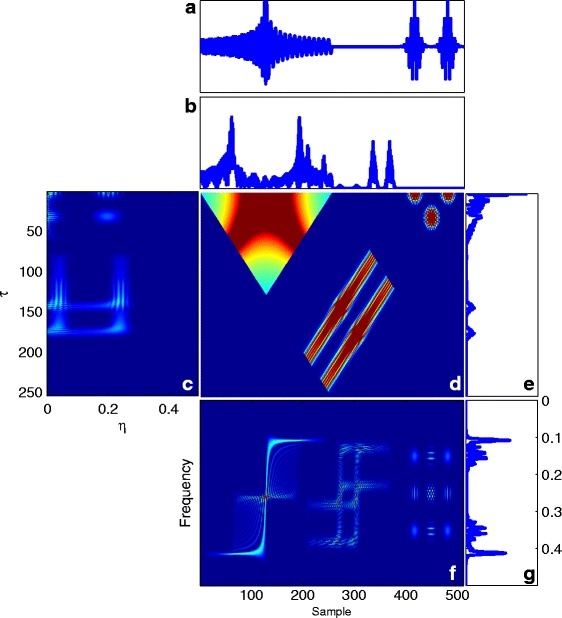
**a** A sample signal *x*(*t*), **b** *S*_*xx*_(*t*) showing the local symmetry of *x*(*t*), **c** the ambiguity plane, **d** the instantaneous auto-correlation, illustrating the similarity of *x*(*t*) with its time lag **e** the auto-correlation function with its maximum peak at *τ*=0**f** the WV transform showing the spectral properties of *x*(*t*) versus spatial samples **g** the spectrum of *x*(*t*)

To illustrate the application of the introduced TF transforms, we consider a sample segment of the signal *x*(*t*)=*y*(*t*) consisting of a chirp and two Gaussian signals [16], shown in Fig. 2. The results of (1)–(6) calculated for this signal are shown in Fig. 2. To understand the significance of TF analysis, compare the spectrum of the signal in Fig. 2 g with the WV distribution of this signal in Fig. 2 f. As we can see, the evolution in time of the frequency content of the signal is totally lost in Fig. 2 g. However, one can trace variations in the spectral content as a function of time (sample) in Fig. 2 f. For example, the chirp is represented by a relatively wide-band signal, whose (normalized) frequency content is decreasing from 0.4 to 0.1 around time points 20 to 200.

### Interpretive signal processing

All practical signal processing algorithms have some intuitive interpretation besides their mathematical formulation. Let us illustrate the idea with a simple example: it is well known that the *inner product* of discrete-time real signals *x*(*t*) and *y*(*t*) is mathematically defined as follows

8 $$ \langle x(t),y(t) \rangle = \sum_{t=-\infty}^{\infty} x(t)y(t)   $$

In (8), whenever *x*(*t*) and *y*(*t*) have the same sign (both positive or negative), a positive value is added to the summation; while when they differ in sign a negative value is added. Therefore, for zero-mean signals (which have both positive and negative values), if the inner product is close to zero, one can conclude that the two signals do not have a similar pattern, while a great absolute value of the inner product is an indication of average “co-variance” or similarity of the two signals. In fact, the multiplication operator in (8), provides a measure of point-wise similarity, while the summation gives the average behavior of this similarity. This basic interpretation has led to the definition of a hand full of other measures of signal co-variance. For instance, one may subtract the mean values of *x*(*t*) and *y*(*t*) to centralize the data (when the mean values do not convey information), or to normalize it by the square roots of the energies of *x*(*t*) and *y*(*t*), in order to make the inner product dimensionless and to normalize it between -1 and +1. For certain applications, researchers have replaced the point-wise product of *x*(*t*)*y*(*t*) by other measures of similarity, like sign(*x*(*t*)*y*(*t*)) (cf. [17]). We can see that while the “inner product as a measure of signal similarity” is a common property of various forms of these definitions, employing the appropriate form hinges on the application.

Herein, we refer to the procedure of reforming signal processing algorithms based on their interpretation, as *interpretive signal processing* (ISP). In [12], we used the notion of ISP to apply *matched filters* in genomic signal processing. We show how this procedure helps us reformulate the Cohen class of time-frequency transforms for genomic sequence data.

### Extending the time-frequency analysis to genomic sequences

As shown in Fig. 1, the core of all bilinear transforms is the instantaneous cross- or auto-correlation. In order to extend TF transforms to non-numeric genomic sequence data, *we propose to replace the product of x*(·) *and y*(·) *in r*_*xy*_(*t*,*τ*) *with the similarity matrix entries of genomic sequences*. This idea is based on the interpretation of the product as a measure of similarity. Since a DNA sequence consists of four nucleotides, Adenine, Cytosine, Guanine, and Thymine, denoted by A, C, G, and T, respectively, a possible choice of the similarity matrix is the identity matrix represented as

9 $$  \begin{aligned} &\qquad\qquad\texttt{A}\;\;\; \texttt{C} \;\;\;\texttt{G} \;\;\;\texttt{T}\\[-3pt] &S = \begin{array}{c} \texttt{A}\\ \texttt{C}\\ \texttt{G}\\ \texttt{T} \end{array} \left(\begin{array}{cccc} 1{\phantom{0}} & 0{\phantom{0}} & 0{\phantom{0}} & 0\\ 0{\phantom{0}} & 1{\phantom{0}} & 0{\phantom{0}} & 0\\ 0{\phantom{0}} & 0{\phantom{0}} & 1{\phantom{0}} & 0\\ 0{\phantom{0}} & 0{\phantom{0}} & 0{\phantom{0}} & 1\\ \end{array} \right) \end{aligned}  $$

which indicates that each of the DNA nucleotides only resembles itself. In practice, based on experimental statistics, a bioinformatician may choose non-zero values for the off-diagonal entries or values smaller than one for the diagonal entries, indicating the probability of base-pair mutation at a specific locus. Moreover, in order to analyze a specific nucleotide and neglect others, all irrelevant entries of the matrix can be set to zero, which results in selective frequency or selective pattern analysis for DNA sequences. For proteins sequences, one may use BLOSUM62 and PAM250 matrices [18].

Since all bilinear transforms contain the product of two terms, the proposed approach is an indirect means of mapping non-numeric sequences to numeric values, which is guaranteed to serve as a similarity measure (by definition), and does not suffer from the *ordering* issue in previous mapping techniques (as noted in the introduction).

## Results

### Case studies

Before applying the method to real DNA and protein sequences, let us consider a synthetic sequence for illustration.

#### A synthetic DNA sequence

For illustration, consider the following synthetic periodic DNA sequence, with period 4 (ACGT) and length 1000.

10 $$ x_{0}[n] = \cdots \texttt{ACGTACGTACGTACGT}\cdots   $$

Real DNA sequences are never fully periodic. In order to make the sequence more realistic, we add some random changes (noise) to the sequence, by random substitutions of some nucleotides.

11 $$ x[n] = \cdots \texttt{AGGTTCGTACGAACCT}\cdots   $$

**Fig. 3:**
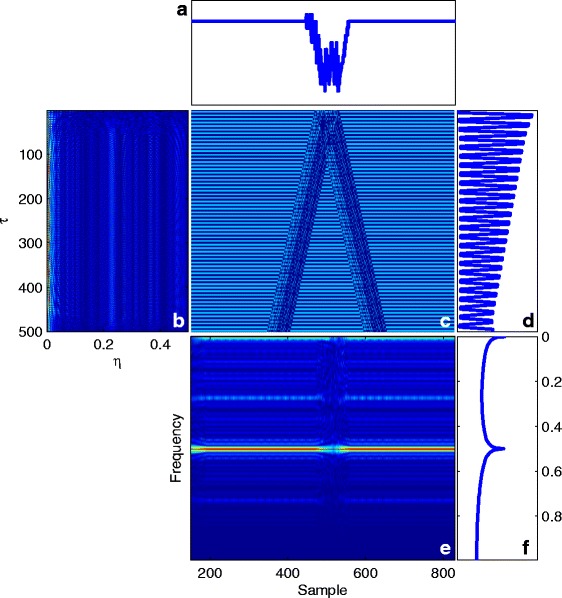
Results of (1)–(6) for the synthetic DNA sequence in (11). **a** The symmetry function using (3). **b** The ambiguity function using (6), showing the stationarity and spectral color of the sequence. **c** The instantaneous auto-correlation using (1), representing the similarity of the sequence with its time lag nucleotides. **d** The auto-correlation of the sequence using (2), with a maximum peak at *τ*=0. **e** The WV transform using (5), indicating the time-frequency properties of the sequence. **f** The spectrum using (4), which shows the global spectral properties for the sequence

Using the trivial similarity matrix in (9), Eqs. (2)–(6) can be calculated for this nucleotide sequence using (1). The results are summarized in Fig. 3.

Part (e) in Fig. 3 shows the WV plane for the noisy synthetic sequence in (11). We can clearly see that the pseudo-periodicity of the sequence has led into a horizontal line at 0.25 (normalized frequency) in this figure, which is equivalent to a periodicity of 1/0.25=4 samples. Also, this pseudo-periodicity causes a peak in part (f), which shows the global spectral properties of the signal. It has been shown that for stationary and temporally correlated signals (i.e., a colored spectrum), most of the ambiguity function’s energy is spread in the *τ* direction around *η*=0 [5]. This explains the ambiguity function form of our synthetic periodic sequence, which is stationary over time. More examples will be shown for real sequences in the next section. The effect of the correlation lag *τ* is seen in Fig. 3 c, where we can see that due to the periodicity of our synthetic sequence, correlations exist between near (small *τ*) and far samples (large *τ*).

#### A real DNA sequence case study

**Fig. 4:**
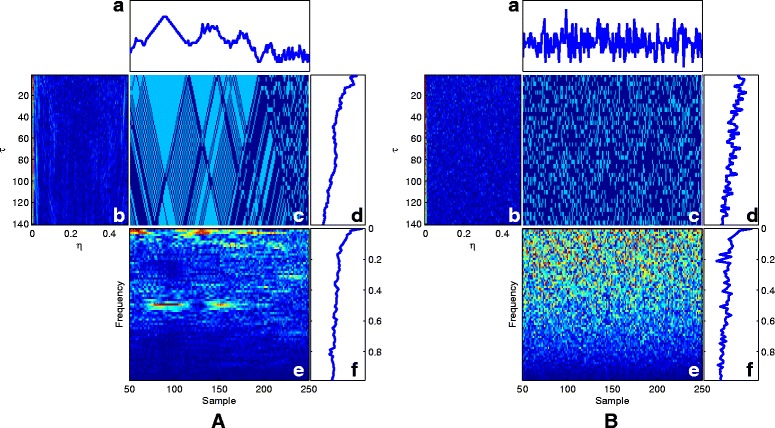
Results of (1)–(6) for **A** the DNA sequence with the accession number FJ807392.1 and **B** a totally random synthetic DNA sequence. The identity matrix is used as the similarity matrix for computing these functions. In each figure: **a** The symmetry function from (3). **b** The ambiguity function from (6) showing the stationarity and spectral color of the sequence. **c** The instantaneous auto-correlation using (1) representing the similarity of the sequence with its time lag nucleotides. **d** The auto-correlation function of the sequence using (2), with a maximum peak at *τ*=0. **e** The WV transform using (5), indicating the time-frequency properties of the sequence. **f** The spectrum using (4), which shows the global spectral properties for the sequence

The proposed framework has been tested on several DNA sequences. As a first case study, we apply the method to a real DNA sequence adopted from the National Center for Biotechnology Information (NCBI) with the accession number FJ807392.1 [19]. Figure 4 illustrates Eqs. (1)–(6) for this real DNA sequence as well as a randomly generated DNA sequence.

For comparison, the results in Fig. 4 A can be compared with similar results obtained from a totally random synthetic DNA sequence of the same length in Fig. 4 B. It is seen that there is no specific structure in the time-frequency transforms of the random sequence, while there are clear structures indicating local periodicities, nonstationarities and spectral color in the real DNA sequence.

**Fig. 5:**
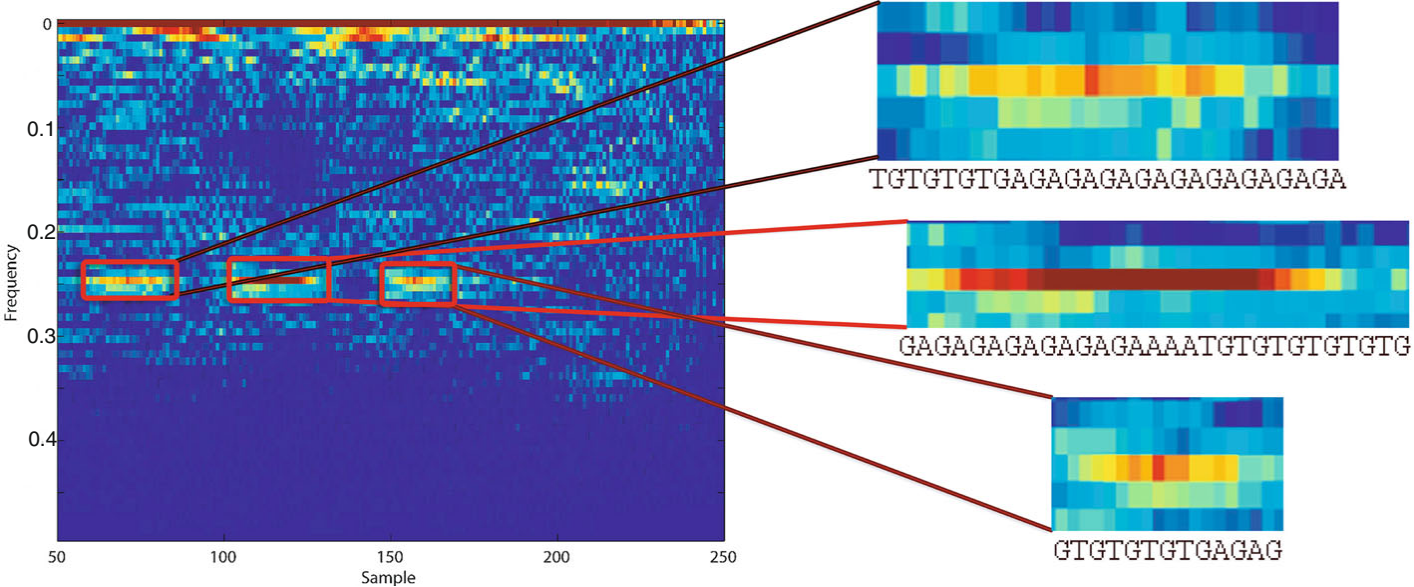
The WV transform for the DNA sequence in Fig. 4 A and its corresponding DNA sub-sequence. Note the local repetitive structure in the DNA sequence and its corresponding pattern in the WV plane

According to NCBI, FJ807392.1 is a *Helice tientsinensis microsatellite* TJH03 *sequence*, which is a repetitive sequence with 282 base nucleotide pairs. Figure 5 is a zoom-in of the WV plane for this sequence, from which its repetitive sub-sequences can be well seen and detected at 0.25 and 0.02 normalized frequencies, corresponding to both short term and long term periodicities in the sequence.

Moreover, due to the repetitive structure of this sequence, it can be considered a stationary and colored sequence, which explains the ambiguity plane structure in Fig. 4 A part b, which is concentrated around *η*=0 and spread in the direction of *τ*. Therefore, the proposed ambiguity plane can be used to study the spectral and stationarity properties of DNA sequences. This is especially useful for feature extraction and classification of DNA and protein sequences.

#### Identification of protein coding DNA regions

As a second case study, the proposed method is compared with a well known method called *indicator sequences*, for analyzing DNA sequences [1, 20]. Accordingly, indicator sequences of a DNA sequence are four binary sequences corresponding to the four different nucleotides. Each sample in the indicator sequence specifies the presence of the nucleotide at that position. The following is an example of a DNA sequence and its indicator sequences:

12 $$ { \begin{aligned} \begin{array}{llllllll} \text{DNA sequence:} &\texttt{A}{\phantom{0}}&\texttt{G}{\phantom{0}}&\texttt{C}{\phantom{0}}&\texttt{C}{\phantom{0}}&\texttt{T}{\phantom{0}}&\texttt{G}{\phantom{0}}&\texttt{A}\\ \text{Indicator sequence}~u_{A}[n]: &1{\phantom{0}}&0{\phantom{0}}&0{\phantom{0}}&0{\phantom{0}}&0{\phantom{0}}&0{\phantom{0}}&1\\ \text{Indicator sequence}~u_{C}[n]: &0{\phantom{0}}&0{\phantom{0}}&1{\phantom{0}}&1{\phantom{0}}&0{\phantom{0}}&0{\phantom{0}}&0\\ \text{Indicator sequence}~u_{G}[n]: &0{\phantom{0}}&1{\phantom{0}}&0{\phantom{0}}&0{\phantom{0}}&0{\phantom{0}}&1{\phantom{0}}&0\\ \text{Indicator sequence}~u_{T}[n]: &0{\phantom{0}}&0{\phantom{0}}&0{\phantom{0}}&0{\phantom{0}}&1{\phantom{0}}&0{\phantom{0}}&0\\ \end{array} \end{aligned}}  $$

**Fig. 6:**
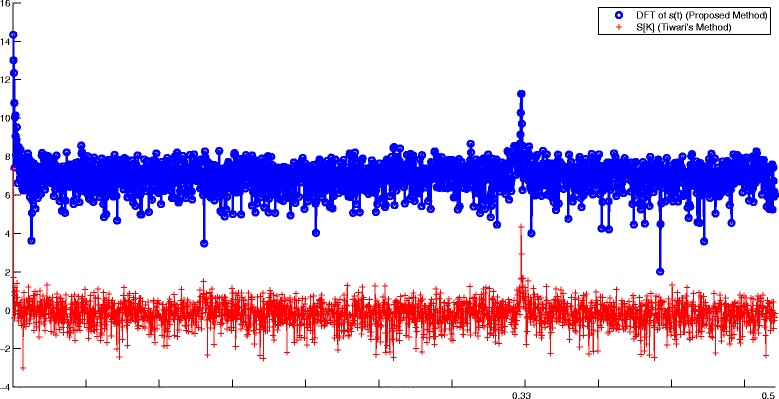
A comparison between Tiwari’s method [20] and the DFT of (3) for a DNA sequence with the accession number NM_001244612.1. Both signals have a peak at *k*=*N*/3 (for better illustration one of the signals has been shifted up)

According to [20], the indicator sequence can be used to define the DNA spectrum, as follows:

13 $$ S[k] = {|U_{A}[k]|}^{2} + {|U_{C}[k]|}^{2} + {|U_{G}[k]|}^{2} + {|U_{T}[k]|}^{2}  $$

where *U*_*i*_[*k*] is the *discrete Fourier transform* (DFT) of *u*_*i*_[*n*] (*i*={A,C,G,T}). In [20], it has been empirically shown that for a coding region within a DNA sequence (a region that can be converted to a protein), Eq. (13) has a clear peak at *k*=*N*/3 where *N* is the DNA sequence length. While this observation has been referred to in various studies, to the authors’ knowledge, no mathematical explanation has yet been presented for it. However, using our proposed machinery, one observes a similar periodicity by using the identity similarity matrix (9) and by calculating the spectral function (4). The results of this comparison are shown in Fig. 6.

To illustrate this, we take a DNA sequence with the NCBI accession number NM_001244612.1. This DNA sequence with 4165 base pairs is known to be a coding region for human proteins. Figure 6 shows the DNA spectrum calculated from indicator sequences. The second signal in Fig. 6 is the Fourier transform of the proposed symmetry function (3). The peak for the Fourier transform of (3) reports a periodicity at *N*/3.

## Discussion

In our experiments, we show that by defining the instantaneous auto-correlation of DNA sequences using similarity matrices, local and global periodicities in the sequences can be detected by the WV transform in (5). Symmetric sub-sequences can be determined by the symmetry function (3) and the stationarity and spectral properties of sequences can be recognized by the ambiguity function (6). Also, the global spectrum of the sequences can be calculated by the spectrum of the sequences (4) and the global correlation of the sequences can be found by the auto-correlation function (2).

The major advantage of ISP *per se* is to process the non-numerical symbols directly (instead of converting the symbols into numerical values). This property simplifies the interpretation of the output of signal processing algorithms when applied to non-numerical symbols. However, ISP is not always trivial, since the interpretation of mathematical equations is not always straightforward. Moreover, the interpretation of signal processing algorithms is not necessarily unique and in some cases unfeasible. Therefore, in practice ISP can result in algorithms that are only partially applicable to non-numerical data while the remaining parts are left unchanged—as in the TF transforms presented in this work, in which only the instantaneous auto-correlation function was replaced with the similarity matrix of genomic sequences.

## Conclusion

In this study, using the notion of interpretive signal processing (ISP), the conventional time-frequency transforms have been extended to analyze non-numerical genomic sequences. Applications of the proposed machinery in determining genome periodicity and detecting tandem repeats were presented using synthetic and real DNA sequences. The results show that the proposed ISP-inspired TF transforms (to which we refer as the interpretive TF analysis) can be useful to analyze genomic sequences.

Other aspects of the proposed interpretive TF analysis that require further work are: 1) investigating other biologically-inspired applications of the proposed machinery; 2) studying different choices of similarity matrices in various applications such as DNA or protein sequence alignment; 3) integrating the proposed machinery in existing sequence analysis toolboxes for extracting further quantitative and visual information from genomic sequences; 4) using the TF representations as features (as an image) and using image classification and clustering techniques for classifying unknown genomic sequences; and 5) extending the hereby proposed notion to higher order spectra (HOS) and higher order time-frequency analysis. These contributions can be considered as a step towards the development of a rigorous mathematical construct for genomic sequence signal processing.

## Abbreviations

AF: Ambiguity function
DFT: Discrete Fourier transform
HOS: Higher order spectra
ISP: Interpretive signal processing
TF: Time-frequency
WV transform: Wigner-Ville transform
